# Association of Veterinary Faculties

**Published:** 1895-10

**Authors:** 


### ASSOCIATION OF VETERINARY FACULTIES.

The Association of Faculties has proven to be one of the strongest movements ever inaugurated. Its harmony, conservatism, and sincere purposes have won for it the admiration of almost every school in North America, and the unqualified support of nearly every school on this continent.

What a grand and impressive sight to see mingling together in a common purpose for the general good of all representatives of Harvard Veterinary Department, New York Veterinary College, Veterinary Department of University of Pennsylvania, National Veterinary College, McKillip Veterinary College, Veterinary Department Detroit School of Medicine, Chicago Veterinary College, Kansas City Veterinary College, Toronto Veterinary College, and the Veterinary Department of the Iowa Agricultural College. This organization has come to stay and be a power in the land.

Professor McEachran's unavoidable absence through official duty was a great disappointment, but his letter, full of ringing sound sentences, earnest, and far-reaching in character, fully demonstrated how intensely interested he is in this grand movement.

President Lyman was more than happy in reporting to the parent association of this offspring of the great good already obtained, and how earnest and interested every member in attendance seemed.

Everyone was glad to see Professor Duncan at the convention, and he was an eager listener and interested attendant at the meetings of the Association of Faculties, to learn on behalf of the Toronto Veterinary College how strong was the intent and purposes of those who are giving so much time and effort to this movement. He went home delighted.

